# Cryptic Early Gastric Carcinoma in Gastric Stump: Challenges in Diagnostic Evaluation

**DOI:** 10.1155/2019/1794370

**Published:** 2019-12-17

**Authors:** Subramanya Sakaleshpura Mallikarjunappa, Shriram Jakate

**Affiliations:** ^1^MD, PGY-1 Resident in Pathology, Department of Pathology, Rush University Medical Center, 1750 W. Harrison St., Suite 570, Chicago, IL 60612, USA; ^2^Professor of Pathology, Gastroenterology and Hepatology, Department of Pathology, Rush University Medical Center, 1750 W. Harrison St., Suite 570, Chicago, IL 60612, USA

## Abstract

Partial gastrectomy, performed for any indication, is a well-recognized risk factor for carcinoma developing in the gastric remnant (so-called “gastric stump carcinoma”). In symptomatic patients with gastro-enteric anastomosis, it is a common practice to endoscopically evaluate the patency and the status of the anastomosis and procure biopsy samples when endoscopic abnormalities are noted. We describe a case with Billroth I gastroduodenal anastomosis with oozing and friability at the anastomosis site which was biopsied. The biopsies showed invasive intestinal-type adenocarcinoma. Subsequent completion gastrectomy showed no grossly visible tumor and required extensive initial and additional sampling of the anastomosis and the surrounding stomach to locate a small focus of invasive adenocarcinoma limited to the mucosa (“early gastric carcinoma”). This case illustrates a known complication of partial gastrectomy and highlights challenges in diagnostic evaluation of early gastric carcinoma after gastrectomy.

## 1. Introduction

The predisposition for gastric carcinoma is variably increased in all cases of partial gastrectomy regardless of the indication of the initial operation (benign condition such as peptic ulcer disease or neoplasms such as gastrointestinal stromal tumor or carcinoma) or the type of partial gastrectomy (distal gastrectomy with Billroth I or II anastomosis or proximal gastrectomy). The risk of carcinoma is apparent after a long period of postoperative latency of several years. Carcinoma developing in gastric remnant or gastric stump carcinoma, is often considered to show poor prognosis due to infiltration of adjacent organs, lymph node metastasis and low resectability. However, gastric remnant may also harbor a small cryptic focus of adenocarcinoma that is incidentally detected when the gastric remnant or the anastomosis is endoscopically sampled. Such early gastric carcinoma poses challenges in diagnostic evaluation when completion gastrectomy is performed, since no tumor or lesion may be grossly visible in the resected specimen. Extensive and repeated sampling and correlation with the prior endoscopic biopsy site may become necessary to locate and evaluate the tumor.

## 2. Case Report

**Figure 1:**
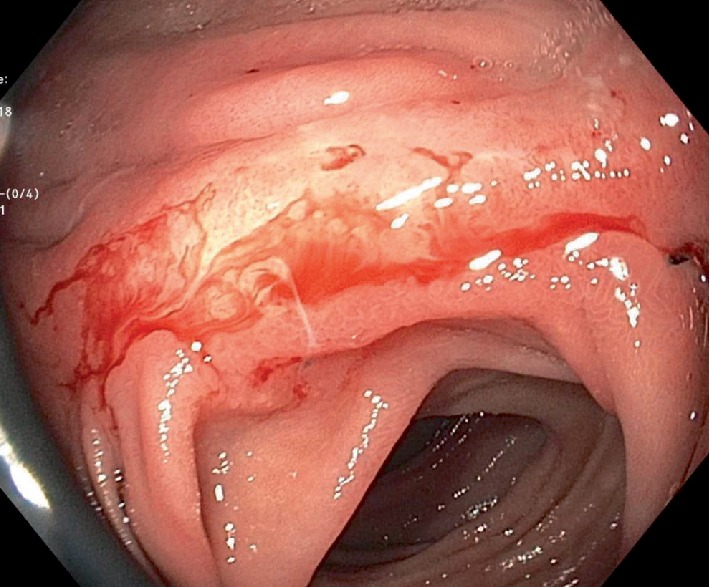
EGD showing patent gastroduodenal anastomosis with oozing, erosion, and friable mucosa.

**Figure 2:**
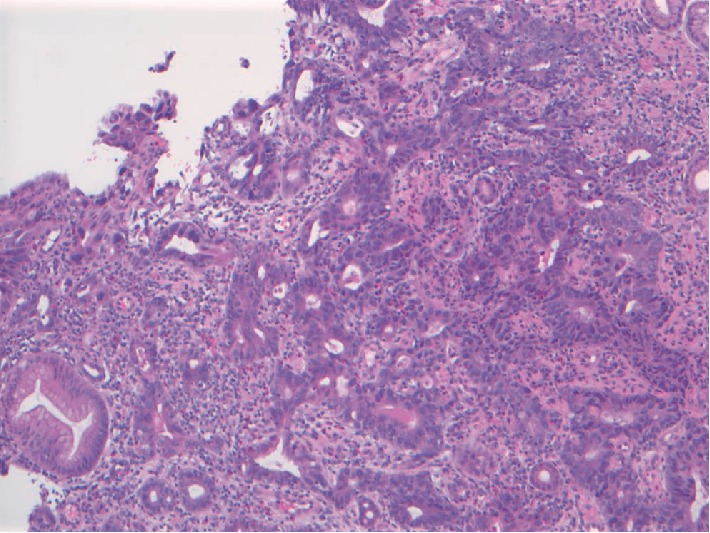
Microphotograph of a biopsy fragment from the anastomosis showing invasive moderately differentiated adenocarcinoma in the gastric mucosa (hematoxylin and eosin stain, ×100 magnification).

**Figure 3:**
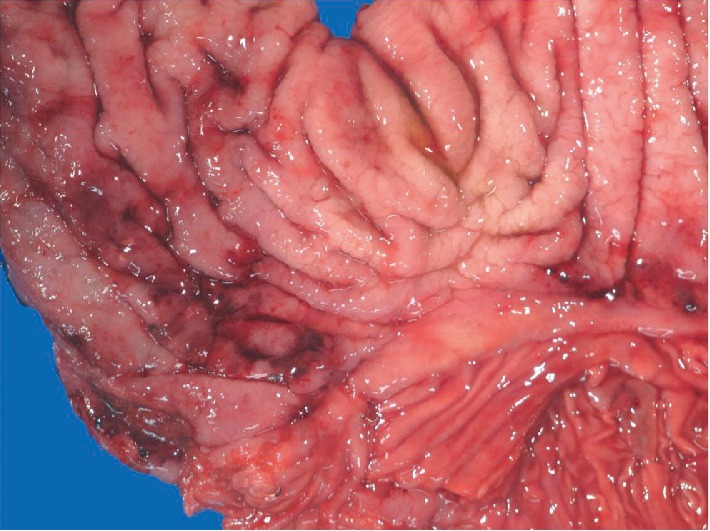
Completion gastrectomy showing remnant stomach (upper two third of the image), attached duodenum (lower third of the image) and intervening gastroduodenal anastomosis. The specimen shows no mass or lesion and only patchy congestion near the anastomosis.

**Figure 4:**
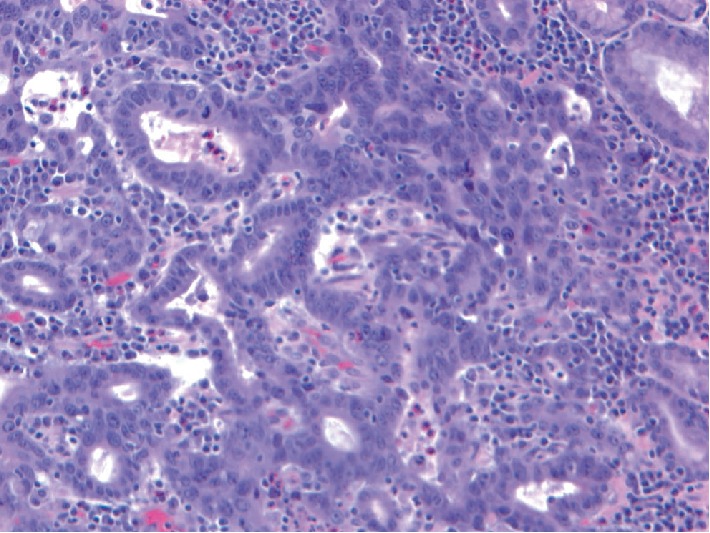
Microphotograph from the additional section from the anastomosis of the completion gastrectomy showing moderately differentiated adenocarcinoma invading the lamina propria of the gastric mucosa (hematoxylin and eosin stain ×200 magnification).

An 86-year-old male patient presented with fatigue and 15 lb unintentional weight loss in 3 months. His past medical history included distal gastrectomy for peptic ulcer disease and Billroth I reconstruction 30 years prior and radical prostatectomy for prostate carcinoma 2 years prior. He also had history of coronary artery disease, hypertension, gastroesophageal reflux disease and smoking (2 packs/day ×40 years). Review of symptoms and physical examination was unremarkable. Laboratory investigations revealed iron deficiency anemia. Esophagogastroduodenoscopy (EGD) and colonoscopy were performed given the symptoms and history of gastrectomy. EGD showed a patent Billroth I gastroduodenal anastomosis with focal erosion, oozing and friable mucosa around the anastomosis (Figure 1) from which biopsies were taken. Two of four biopsy pieces from the anastomosis showed gastric mucosa with invasive moderately differentiated adenocarcinoma, intestinal-type (Figure 2). Immunostains supported a gastric primary (positive staining for cytokeratin 7, cytokeratin 20, and CDX2) and ruled out metastasis from prostate (PSA negative). Immunostains for mismatched repair proteins (MLH1, MSH2, PMS2, and MSH6) showed retained expression consistent with microsatellite stability (MSS). Colonoscopy showed a small solitary 5 mm tubular adenoma in the sigmoid colon. Staging CT showed no gastric wall thickening and no regional lymph node enlargement or metastasis. Five weeks after EGD, completion total gastrectomy was performed. On gross examination, the resected specimen showed no tumor mass or lesion, and only patchy congestion near the anastomosis (Figure 3). Initially, 20 sections were taken randomly from different areas of the stomach, gastroduodenal anastomosis and the attached duodenum. However, apart from moderate chronic gastritis and bile reflux gastritis, no intestinal metaplasia, dysplasia, or carcinoma was identified. There were no Helicobacter organisms. The prior endoscopic biopsies were reviewed again, confirming the malignant tumor. The completion gastrectomy specimen was again sampled with additional 15 sections taken exclusively from the nodular anastomosis site (the location of the original biopsy sample). This time, 2 of the additional 15 sections showed small foci of invasive moderately differentiated adenocarcinoma with invasion limited to the lamina propria of the mucosa consistent with early gastric carcinoma (Figure 4). No invasion of the underlying submucosa or muscularis propria was present. No lymph nodes were present in the specimen. The gastric stump carcinoma stage was thus pT1aNxMx. All margins (proximal, distal and radial) were negative. The patient has had follow-up of 6 months after gastrectomy with no recurrence or metastasis.

## 3. Discussion

Gastric carcinoma remains the second-most common cause of cancer-related mortality world-wide [1]. The risk factors for gastric carcinoma include male gender, advanced age, *Helicobacter pylori* infection, cigarette smoking, EBV infection, alcohol abuse and CDH1 mutation [2]. Prior partial gastric resection or gastric stump or remnant is an additional well-recognized risk factor and constitutes 1.1–7% of all gastric cancers [1]. Gastric stump carcinoma was originally defined as gastric cancer arising from the remnant more than 5 years after distal gastrectomy for benign disease. However, the definition is now expanded to include prior distal [3] as well as proximal gastrectomy [4] performed for benign [5] as well as malignant conditions [3]. Additionally, in a large population-based study, increased gastric cancer risk in gastric stump was seen only after latency period of 30 years [5]. The pathogenesis of gastric cancer development in the gastric remnant is mainly attributed to gastroduodenal or biliary reflux, mostly after Billroth I procedure [6]. Both exogenous and endogenous factors such as achlorhydria, hypergastrinemia and biliary reflux, Epstein-Barr virus and *Helicobacter pylori* infection, atrophic gastritis, and also some polymorphisms in interleukin-1*β* and maybe cyclo-ogenase-2 appear to be involved in the etiopathogenesis of gastric stump carcinoma [7]. Gastric stump carcinoma was considered to have poor outcome [8], but studies have shown that there appears to be no difference in the prognosis between primary gastric carcinoma and stump carcinoma [6, 9]. To improve the prognosis of gastric stump carcinoma, endoscopic surveillance of gastric remnant and anastomosis site for detection of early gastric carcinoma is recommended [8, 10]. Such surveillance is best commenced 15 years after the original surgery with multiple biopsies of the gastroenterostomy [11]. The sampling protocol from the anastomosis should be similar to the environmental metaplastic atrophic gastritis, consisting of about 8–12 targeted biopsies [12].

Early gastric carcinoma (EGC) is defined as carcinoma invading up to the submucosal layer regardless of nodal metastasis. EGC is heterogeneous in location, endoscopic features, histology, nodal metastasis and prognosis [13]. EGC, found during endoscopic biopsies, may reveal nonneoplastic pathology result after complete removal due to incorrect localization or pathological overestimation of dysplasia [14]. Hence, review and confirmation of invasive carcinoma from endoscopic biopsies may be required. Additionally, since EGC may be grossly poorly apparent, several sections from resected specimen may be needed to locate and evaluate EGC.

Our patient displays multiple well-documented features including risk factors such as advanced age, male gender, long history of cigarette smoking, gastric stump of 30 years and EGC at the anastomosis. Furthermore, pathologically, this case illustrates the need for review of prior biopsies to confirm invasive carcinoma and repeated sampling of the anastomosis from the gastrectomy specimen.
